# Metasurface-Based Phosphor-Converted Micro-LED Architecture for Displays—Creating Guided Modes for Enhanced Directionality

**DOI:** 10.1021/acsnano.4c13472

**Published:** 2024-12-23

**Authors:** Debapriya Pal, Toni López, A. Femius Koenderink

**Affiliations:** † Department of Physics of Information in Matter and Center for Nanophotonics, NWO-I Institute AMOLF, Science Park 104, NL 1098XG Amsterdam, The Netherlands; ‡ Lumileds Germany GmbH, Philipsstr. 8, D-52068 Aachen, Germany

**Keywords:** LEDs, phosphor, guided, LDOS, metasurface, plasmonics, Fourier

## Abstract

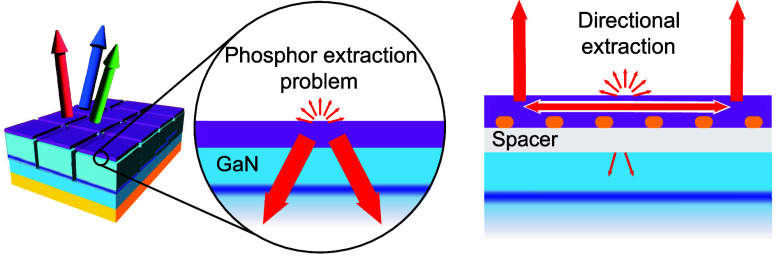

Phosphor-converted micro-light emitting diodes (micro-LEDs) are a crucial technology for display applications but face significant challenges in light extraction because of the high refractive index of the blue pump die chip. In this study, we design and experimentally demonstrate a nanophotonic approach that overcomes this issue, achieving up to a 3-fold increase in light extraction efficiency. Our approach involves engineering the local density of optical states (LDOS) to generate quasi-guided modes within the phosphor layer by strategically inserting a thin low-index spacer in combination with a metasurface for mode extraction. We demonstrate the trade-offs between blue light pumping, LDOS enhancement at the converted emission wavelength, and radiation pattern control using a stratified system solver for dipole emission. Experimentally, the integration of plasmonic antennas and a silica spacer resulted in a 3-fold overall brightness enhancement, with nearly a 4-fold increase in forward emission. This nanophotonic metasurface waveguide design is a critical advancement for producing bright, directional micro-LEDs, particularly in augmented/virtual reality (AR/VR) devices and smartwatch displays, without the need for bulky secondary optics or reflectors.

Micro-light emitting diodes (micro-LEDs) have emerged as next-generation high-performance displays for devices like smartphones, wearables, and head-mounted augmented and virtual reality (AR-VR) gadgets.^1−3^ Micro-LEDs’ advantages for displays include superior brightness, high contrast ratio, reliability, luminous efficiency, and fast response time.^3−5^ High-resolution displays demand densely packed pixels in chips with high pixel density, typically with less than 50 μm^6^ pitch, as seen in micro-LED arrays. Micro-LED displays can be classified into direct view and indirect view^7^ applications. Direct-view applications, such as monitors and advertising signages, require isotropic emission with a Lambertian profile for visibility from all angles. In indirect view applications, such as AR-VR smart glasses and navigation windshields, light is waveguided, processed, and precisely projected to form the final display image visible for near-eye view. Micro-LED displays can achieve a full-color gamut through either direct or color-converted emission processes. The direct emission approach involves assembling discrete native micro-LEDs, each emitting one of the three primary colors, to form an RGB (red, green, blue) full-color pixel.^8−12^ This method has drawbacks, such as the low external quantum efficiency of red InGaN LEDs and challenges in fabrication, miniaturization, and the potential for defects. Color-converted micro-LED technology uses phosphor materials to address these challenges by incorporating only a single highly efficient UV (ultraviolet)^13,14^ or blue^15,16^ micro-LED, simplifying the chip-level production process. The phosphor layer absorbs blue pump photons and re-emits them at larger wavelengths. Advanced narrow-band phosphor materials^17,18^ like quantum dots,^19−21^ perovskites,^22−24^ and photostable dyes^25−27^ have significant industrial potential, offering bright emissions, high absorption, tunable bandgaps, large Stokes shift, and exceptional color purity. Ongoing research in phosphor materials is focused on achieving strong absorption in the blue spectrum, exceptional quantum efficiency, and photostability under harsh illumination conditions, typically at W mm^–2^ levels,^16,28,29^ highlighting the major challenges in these areas. Partial color conversion with blue micro-LEDs may affect red and green color purity, while full conversion with UV micro-LEDs depends solely on phosphor emissions. Despite the increased complexity in the phosphor printing, encapsulation layer, and the possible need to include optical filtering to improve color purity, the performance and yield benefits of full-color conversion are anticipated to outweigh the cost savings of the native RGB approach. In micro-LED display arrays, the phosphor must be placed directly on top of the blue die chip^30−32^ in individual LEDs to prevent optical cross-talk from scattering effects and meet the aspect ratio requirement (typically 1:10 for thickness to base dimension). Additionally, achieving directional emission without bulky reflectors or secondary optics is critical for indirect, near-view devices like AR/VR and for select direct-view applications like smartwatches.

Nanophotonics offers solutions to address some of these challenges. Diffractive 2D arrangements of plasmonic or dielectric nanoscatterers placed in (sub) micron-thickness phosphor films are effective in enhancing light coupling, accelerating emission through the Purcell effect, and controlling angular radiation patterns in remote-phosphor architecture (phosphor far away from blue die) for general-purpose LEDs applications.^33−37^ Typical strategies in nanophotonics to enhance the absorption of blue pump light and improve converted emitted light extraction in specific directions involve two carefully tuned effects. First, the emissive layer is generally engineered to act as a waveguiding layer in which the guided mode dominates the local density of optical states (LDOS), thereby promoting emission into the waveguide. This generates spatial coherence,^38,39^ as the emission occurs mainly in specific in-plane momenta associated with the waveguide dispersion. The second effect involves outcoupling this waveguided emission into defined far field directions by diffractive resonances created by periodic arrays of scatterers.^40^ Typical photoluminescence enhancements of order 10–12 are achievable, resulting from typically up to 5-fold pump enhancements and up to 3-fold improvements in extraction efficiency.^41,42^ In the seminal work by Lozano et al.,^34^ a remarkable 60-fold enhancement was achieved, driven by a 10-fold improvement in extraction efficiency, although limited to specific wavelengths and directions. Unlike general-purpose LEDs, display micro-LEDs pose even more significant challenges to blue pump light absorption and converted emission extraction efficiency. Traditional nanophotonic techniques cannot be directly applied to display micro-LEDs because the phosphor must be put directly on top of the blue LED chip (approximately refractive index of 2.4 for the top GaN layer) to prevent leakage and meet size constraints. This high index causes the phosphor layer to lose its waveguiding property, leading to most of the phosphor emission being directed toward GaN, posing a significant challenge for light extraction (see Figure 1a,b).

**Figure 1:**
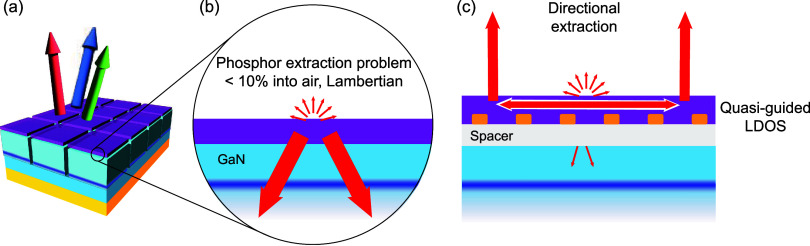
Concept sketch of the current state of the art and our proposal. (a) Schematic of a proposed phosphor-converted microLED display array using InGaN/GaN, featuring a submicron-thick phosphor layer on a thin-film blue LED chip. Such architectures typically include a 6–10 μm GaN layer, InGaN multiple quantum wells, an approximately 100 nm layer of p-GaN, a conductive ITO layer, and a backside metallic mirror. (b) Close-up showing that most phosphor emission will be directed into the blue LED, with only 10% emitted into air in a Lambertian profile. (c) Inserting a spacer between the high index GaN and phosphor enhances emission into quasi-guided LDOS within the phosphor, while corrugation facilitates directional extraction.

Two critical questions for nanophotonic improvement of phosphor-based micro-LEDs are (i) Can we replicate the waveguide mode characteristics of the phosphor at the converted emission wavelength without obstructing the blue pump photons from the LED? and (ii) How does the physics of diffractive metasurfaces for outcoupling interplay with the high index contrast stratified geometry of the LED-phosphor combination? For instance, can plasmonic surface lattice resonance strategies for directional outcoupling remain effective and directly transposed to stratified architectures? In this work, we tackle both of these challenges. Our proposal involves specific geometrical architectures where the phosphor is concentrated in submicron-sized layers and is separated from the blue die chip by either (a) a micron-sized dielectric spacer with a refractive index lower than that of the phosphor (referred to as low index normal dielectric spacer) or (b) by a 1D dielectric multilayer stack of materials with alternating refractive indices (Bragg stack spacer). We present rigorous theoretical calculations to design the spacers that promote emission into the guided modes. We perform experiments demonstrating the advantages of inserting the proposed thin low-index spacer combined with periodic corrugation to facilitate the outcoupling of guided mode emissions (see Figure 1c). Notably, the emission is highly directional toward the air, resulting in a 4-fold enhancement in the forward direction compared to a planar layer of the dye-doped polymer layer of similar thickness directly on top of GaN. The paper is structured as follows: first, we discuss emission behavior in stratified systems without periodic corrugation, using multilayer theory for the LDOS and radiation patterns and evaluate the trade-offs between capturing emission into the waveguiding phosphor layer on the one hand and efficiently pumping the phosphor on the other hand. Next, we explore the inclusion of a diffractive plasmonic metasurface for outcoupling and present experimental Fourier-microscopy data that quantify the enhanced directional outcoupling of luminescence via the waveguided mode into free space. The proposed solution will open avenues to integrate nanophotonic strategies in complex micro-LED environments to create brighter and more directional displays for compact wearable devices with limited energy capacity.

## Results and Discussion

### Theoretical Design

In this section, we examine the emission physics of a stratified system comprising a GaN blue LED die in close proximity to a phosphor layer. Our design objective is to insert a layer stack between the GaN and the phosphor layer to recover a waveguide mode within the phosphor layer at the phosphor emission wavelength while preserving efficient coupling of blue pump photons from the GaN to the phosphor. The intended waveguide mode should significantly contribute to the local density of states (LDOS). The rationale is that the fraction of converted emission into the waveguide mode offered by the phosphor can ultimately be efficiently outcoupled using periodic corrugation while minimizing emission loss into the GaN die. Although the realization of such a (quasi)-guided mode is not inherently difficult, the related trade-off with respect to blue pump light absorption is nontrivial, as promoting the waveguide mode in the phosphor requires protection against leakage by interspersed low-index spacers. Consequently, this safeguard will directly prevent blue photons generated in the GaN chip from coupling into the phosphor layer, leading to total internal reflection of blue light back into the LED die. To evaluate the trade-off in practical scenarios, we must calculate the fractional LDOS contributions for converted emission into the GaN die, the waveguide modes, and the surrounding air. We also need to calculate the absorption of blue pump light in the waveguiding phosphor layer for the same geometries and evaluate the overall photon budget of useful extracted color-converted emission.

We use the approach developed by Chance, Prock, and Silbey^43^ and Amos and Barnes^44^ to calculate the local density of optical states in stratified layers. The local density of optical states (LDOS) counts the number of electromagnetic modes available at a specific point in space within the frequency ω and ω + dω. To calculate LDOS, we express the required imaginary part of the Green’s function at the source location as a Sommerfield integral over all parallel wave vectors. The modification of the decay rate for an ensemble of randomly oriented dipoles at a fixed height *z* within a layer of thickness *d* in a stratified system can be expressed as^44^
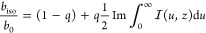
1where *q* represents the quantum efficiency of the emitter and b_0_ is the fluorescence decay rate (radiative plus nonradiative) in the absence of any interfaces. The integrand  quantifies the back-action of the fields emitted by the source and reflected back to the source through multiple reflections. While the integrand has a straightforward form for a single or double interface system, it requires a more involved multilayer calculation for a general stack. According to Amos and Barnes,^44^ for an isotropically oriented ensemble of emitters, the integrand reads
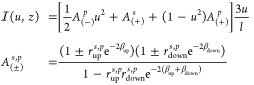
In this context, the integration variable *u* represents the magnitude of the in-plane wave vector *k*_∥_ in the emitter embedding layer, normalized to the wavenumber in free space. The auxiliary variable  relates to the perpendicular component of the momentum. The entire multilayer stack is accounted for by the compound Fresnel coefficients *r*_up_ (from the source layer upward through all layers to the superstrate) and *r*_down_ (traversing all the layers from the source down to the substrate). The exact dipole position within its embedding layer of height *d* is taken into consideration through the phase factors β_down_ = *zl* and β_up_ = (*d* – *z*)*l* toward the bottom and top interface (source at height *z* in the layer). Notably, the integration runs over all positive values of *u*, which contains both propagating, guided, and evanescent contributions. The contributions with *u* below the refractive index of the cladding (air and substrate) correspond to propagating waves denoted as radiated modes, while LDOS contributions from evanescent fields appear at larger *u*. In particular, guided modes manifest as poles of the integrand at values of *u* equal to the mode indices.^45,46^ We have implemented the required ability to solve the stack reflection/transmission coefficients for any parallel momentum, including for evanescent incident waves, using the S-matrix algorithm.^47^ We apply the complex contour integration technique of Paulus and Martin to handle the poles at the guided modes and sub/superstrate light lines.^48^ This allows us to quantitatively calculate LDOS and dipole radiation patterns for dipoles in any stratified system, separating the emission branching into the GaN substrate, the air superstrate, and the guided modes of the system.^45^

**Figure 2:**
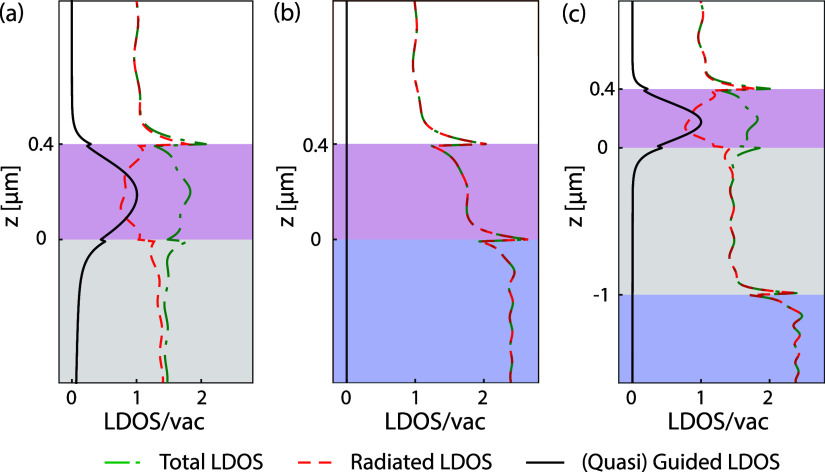
Calculated local density of optical states (LDOS) in stratified systems: (a) phosphor on glass, (b) phosphor on GaN, and (c) phosphor separated from GaN by a glass spacer, all with an air superstrate. Total LDOS (green dash-dotted curves) is separated into radiated (red) and (quasi-)guided (black solid curves) contributions, plotted as a function of emitter position (*z*, perpendicular to interfaces) for an emission wavelength of 0.60 μm, averaged over all dipole orientations. Gray, pink, blue, and white shading indicates glass, phosphor, GaN and air.

Figure 2 reports the main physics of the LDOS as pertinent to phosphor layers in simple stratified geometries. For the calculations, we assume a refractive index *n*_ps_ = 1.75 and thickness 400 nm illustrative of a high index polymer acting as an emissive waveguide layer (parametric dependence further discussed below). We use the refractive indices of GaN (*n*_GaN_ = 2.4)^49,50^ and silica (*n*_silica_ – 1.46)^51^ assuming them as dispersionless and choose the converted emission wavelength as 600 nm to effectively mimic the central wavelengths of both the red (626 nm) and green (530 nm) LEDs,^49^ ensuring the validity of the results across the entire emission spectrum range of phosphor in general. Figure 2a–c shows the orientationally averaged LDOS versus position for (a) the simple system of air-phosphor-glass, (b) phosphor directly on GaN, and (c) the phosphor separated by a thin silica layer from GaN. We separate the total LDOS (green dash-dotted) into radiated (red dashed) and (quasi)-guided (black solid) contributions. Generally, in a medium of index *n* and near a partially reflective interface, the LDOS normalized to that in vacuum oscillates around *n* due to interference as first measured by Drexhage^52^—these Friedel oscillations in LDOS tail off rapidly with distance. At the transition from one interface to the next, discontinuities in LDOS appear due to the discontinuity of the out-of-plane component of the electric field, which is inherited by the LDOS for dipoles oriented perpendicular to the interface. Importantly, for a simple phosphor layer on glass, the LDOS contribution of the guided mode dominates in the phosphor layer, while the contribution of modes that radiate into the far field is suppressed. Even with this example’s modest index contrast, over half of the emission is funneled into guided modes. This slab configuration has a single TE and a single TM-polarized mode. The TE mode dominates the guided mode LDOS, which almost exactly traces the TE guided mode |*E*|^2^ profile. This is the contribution harvested into free space by plasmon surface lattice resonances in recent demonstrations of strongly enhanced and directional light extraction.^34,53^Figure 2b highlights that once high-index GaN replaces the low-index glass substrate, there is no guided mode contribution to the LDOS. By inserting a silica spacer layer, a quasi-guided LDOS contribution is recovered in the phosphor layer, as shown in Figure 2c (refer to the Methods section for the calculation of quasi-guided LDOS). It has the same spatial profile near the phosphor as in the simple phosphor-glass system. The thickness of the separating oxide layer determines the leakage rate and, hence, the strength of the quasi-guided mode contribution, which is reduced compared to the truly guided mode contribution in (a). This calculation showcases that incorporating a low-index spacer between phosphor and GaN recuperates the guided modes of the phosphor as a necessary ingredient for improved light extraction by a diffractive metasurface.

**Figure 3:**
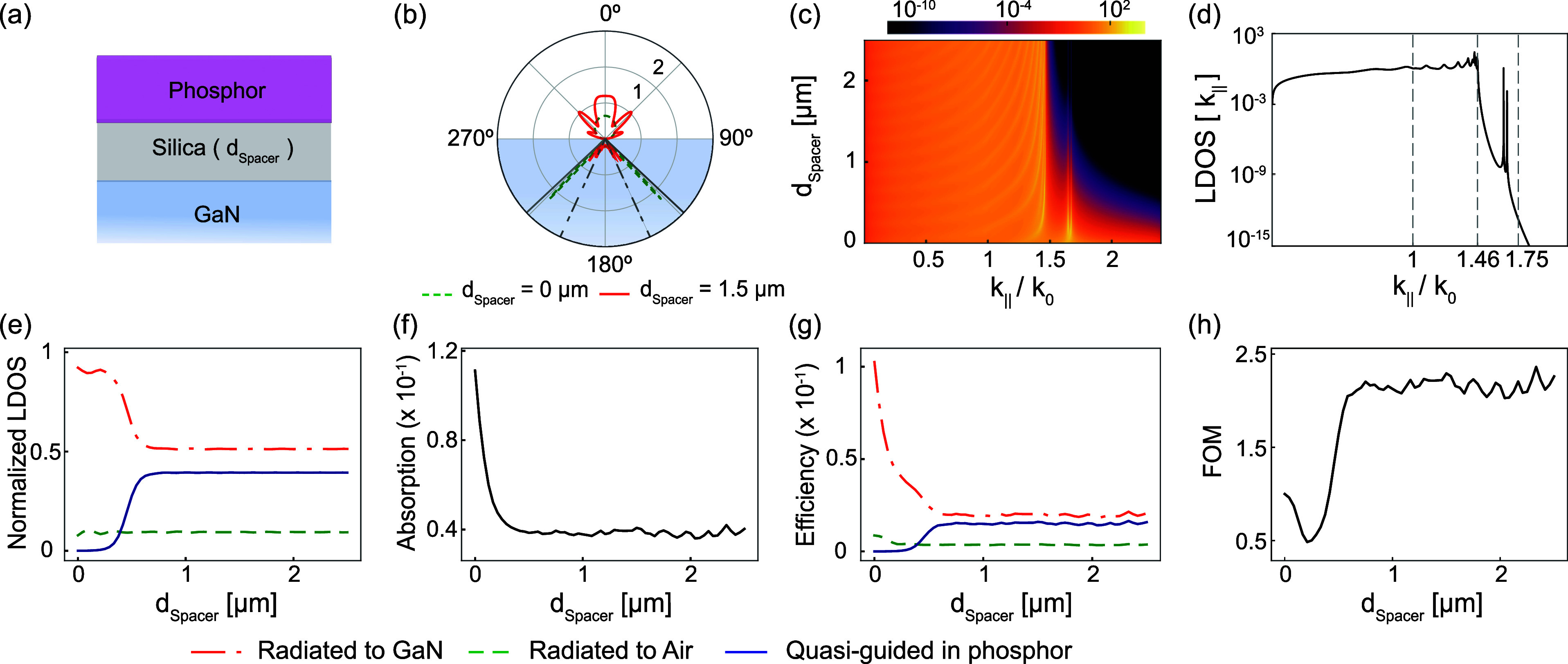
Theoretical analysis of trade-offs in LDOS enhancement, radiation pattern, and pump efficiency for phosphor on GaN when using a low-index silica spacer to protect quasi-guided modes. (a) Sketch of the system, comprising GaN (substrate, *n* = 2.4), a silica spacer (*d*_spacer_ thick, *n* = 1.46), phosphor (0.4 μm thick, *n* = 1.75), and air (*n* = 1) as superstrate. (b) Radiation pattern (dipole orientation averaged) with no spacer (green dashed curve) and 1.5 μm spacer (red solid curve). Upper hemisphere (toward air) is magnified 30× for clarity. Gray solid and dash-dotted lines denote the air–GaN and phosphor–GaN light lines, respectively. (c) Emitted power (dipole orientation averaged) for dipole at phosphor midheight plotted as a function of the in-plane wave vector  and spacer thickness. (d) Crosscut of (c) at spacer thickness *d*_spacer_ = 1.5 μm. As a function of the spacer thickness, plots of (e) normalized emission LDOS contribution of the phosphor layer into various channels, (f) absorption of blue photons in the phosphor layer, (g) efficiency of blue-to-red conversion into different channels, (h) figure of merit (FOM). In panels (d) and (f), red dash-dotted, green dashed, and blue solid curves represent the radiation to the GaN side, airside, and quasi-guided into the phosphor layer, respectively.

Next, we turn to the trade-offs between guided mode LDOS and radiative LDOS, as well as extraction into the air and the substrate as a function of spacers of different thicknesses. Figure 3a shows the far-field radiation pattern as a function of polar angle for dipole emission from the middle of the phosphor layer and isotropically averaging over dipole orientation. When the phosphor is directly placed on GaN without any spacer layer, the emission (green dashed curve) strongly peaks into the GaN and occurs at angles near the GaN–air total internal reflection angle. Via insertion of a spacer layer (red solid curve) (here, e.g., a constant spacer thickness of 1.5 μm), the emission into the GaN strongly reduces as emission into the quasi-guided waveguide mode is promoted. To gain a mechanistic understanding, we study the wavevector resolved LDOS, which is the integrand *I* explained in eq 1 as a function of the normalized in-plane wave vector *u* or  and thickness of the silica spacer. Figure 3b establishes the following main physics. First, the range *k*_∥_ < *n*_air_*k*_0_ corresponds to emission in the upper and lower halfspace at small parallel momenta that fit within the light line of the air superstrate. As seen in panel a, most of this emission is directed upward into the air due to the strong reflection at the GaN–silica interface. The wavevector-resolved LDOS in this regime shows standing wave resonances, typical of the LDOS in an etalon. The range *n*_silica_*k*_0_ (1.46) < *k*_∥_ < *n*_phosphor_*k*_0_ (1.75) corresponds to light that may propagate in the phosphor layer. Here, the LDOS contributions are dominated by two sharp lines, which occur at the guided mode indices of the TE and TM waveguide modes (Figure 3d). At finite spacer thickness, the modes are leaky and, therefore, present a finite width, with the quality factor improving exponentially with increasing spacer thickness as the spacer decouples the guided modes from leaking into the GaN. For infinite spacer thickness, these are poles in the LDOS integrand. Finally, at *k*_∥_ > *n*_phosphor_*k*_0_, there are no LDOS contributions except at very small spacer thickness. The only emission channels for wave vectors above the guided modes are through modes that propagate into the GaN but are evanescent in the spacer, phosphor, and air. In Figure 3e, we calculate the fraction of emitted power lost in various channels by normalizing it to the power emitted in free space and isolating the various channels by integrating over selected *k*_∥_ regions. For the reference case with no spacer between GaN and phosphor, only 8% of emission goes into the air (green dashed curve), whereas 92% of emission is lost into the GaN (red dash-dotted curve) with no guided part contribution (blue solid curve). With an increase in spacer thickness, there is a corresponding increase in emission into the quasi-guided modes within the phosphor layer, accompanied by a reduction in emission toward GaN, with almost no change in the fraction of emission escaping into the air. For a spacer thickness of around 1.5 μm thickness, the emission contribution toward GaN is reduced from 92 to 51%, and the fraction of emission into the air side remains around 10%, while the quasi-guided mode captures up to 39% of emitted photons. This 39% signifies the potential extra emission available for extraction with an embedded periodic metasurface.

Low index spacers between the GaN LED die and phosphor help to recover the guided mode as a resource for light extraction, but that comes at the cost of reducing the absorption of blue light in the phosphor. In a microLED scenario, blue light is generated in multi-quantum wells (InGaN MQWs) inside the GaN with a wide wave vector distribution. Without the spacer, the high parallel momenta in GaN are evanescently coupled to the phosphor. In the presence of the spacer, these wave vectors are totally internally reflected at the GaN–spacer interface, thus not reaching the phosphor. To quantify the pump physics, we use the multilayer S-matrix to calculate absorption in the phosphor, assuming that the MQWs inside the GaN die chip are an angle-isotropic source of blue pump light into bulk GaN. In this analysis, we do not consider the fact that, in practical devices, the top GaN layer has finite thickness with the top GaN surface roughened for improved blue pump light extraction and also, the LED die has an engineered backside, such as a mirror for recycling blue light, which has the potential to improve pump light absorption. The objective here is to optimize the performance in the context of integrated photonic elements,^54−56^ for which analyzing single-bounce interactions is sufficient. However, to fully understand and optimize overall device performance, the effects of light recycling and these practical considerations will need to be addressed in future work.

The absorption cross sections of light-emitting material candidates for phosphors are in the order of 10^–14^–10^–17^ cm^2^,^25−27^ which at typical concentrations (e.g., volume fractions of around 20% in a quantum dot scenario) leads to absorption coefficients of around 10^–1^–10^–3^ in refractive index units. We perform blue pump absorption calculations at 450 nm, pertinent to the usual InGaN MQWs emission wavelength^57^ and assume the absorption coefficient of the phosphor as 0.01. For the reference case, i.e., in the absence of the silica spacer, only ca. 11% of blue photons are absorbed, as seen in Figure 3f. The absorption is reduced by circa a factor of 2.7 with the insertion of finite spacer thickness (roughly 4% of blue pump photons are absorbed only).

The conversion of blue-to-red light depends on the trade-off between the reduced pump absorption and the gain in recuperable red emission. Figure 3g presents a comprehensive system analysis of the expected conversion from blue to red photons. When no spacer separates phosphor and GaN, only about ≲1% red photons are extracted into the air for each blue pump photon available in the GaN. This is significantly lower than the photon generation rate, which is approximately 11% of blue photons absorbed in the phosphor. The reason for this is that 92% of the red emission goes back into the GaN. When a spacer is introduced, the likelihood of a photon being emitted toward the air, GaN, and the guided mode changes significantly. The converted photon flux lost into GaN would be reduced by about 4.9 times, and the direct emission into the air would be reduced by a factor of 2.2, but the conversion efficiency for emission into the phosphor-guided mode would be around 2%. Since this value is twice as high as the direct emission into the air in the reference case, there is a potential 2-fold gain in brightness if the guided mode could be effectively extracted. We have defined a figure of merit (FOM) to evaluate the potential benefits of inserting a spacer in the microLED architecture. The FOM value is the sum of direct radiation into the air and into the guided mode (assuming it could all be extracted), normalized against the reference architecture (no spacer). In Figure 3h, the FOM is plotted as a function of the spacer thickness. Initially, the FOM decreases due to the reduction in pump efficiency, but it quickly recovers due to the creation of the quasi-guided mode. For spacer thicknesses greater than 1 μm, there is a performance boost of approximately 2.3 times compared to the reference case. We note that the dependence shows fringes at large separation due to thin film interference. To summarize the net energy balance, for every 100 blue photons in GaN, with no spacer, only ∼11 converted red photons are generated (assuming unity quantum yield), but fewer than 1 photon (only 9%) is extracted into the air, with the rest lost in GaN. Introducing a spacer reduces red photon generation to ∼4 for every 100 blue photons, with 10% (0.4 photons) radiating directly into air and 40% (1.6 photons) coupled into guided modes, significantly reducing losses in GaN. This 2.7× reduction in absorption is offset by a potential 5-fold increase in extraction efficiency. Reaching this potential would require fully extracting the guided mode through a metasurface. Performance could be further improved through engineering the blue LED to be directive instead of Lambertian and by near-field enhancements of pump light in the phosphor to boost absorption.

**Figure 4:**
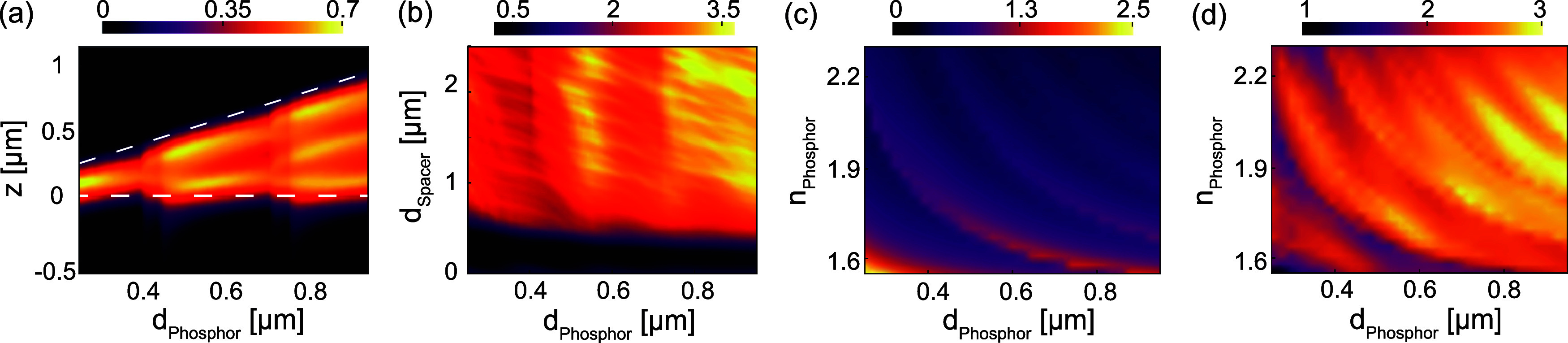
System performance for diverse phosphor choices and spacer configurations. (a) Fraction of emission coupled into guided LDOS within the phosphor layer as a function of emitter position (*z*) and phosphor thickness (*d*_Phosphor_) for an air–phosphor–glass system. For the remaining panels, the layer configuration is identical to that shown in Figure 3, where the phosphor is separated from the GaN by a silica spacer. (b) Figure of merit (FOM) as a function of phosphor and silica spacer thickness. For varying phosphor thickness (*d*_Phosphor_) and refractive index (*n*_Phosphor_), assuming 80% of guided LDOS is extracted (c) minimum spacer thickness needed to achieve this, and (d) corresponding potential figure of merit.

We generalize the calculation example we just walked through in detail above to explore the potential benefits of controlling quasi-guided mode LDOS, thereby gaining insights into the essential design principles and the dependence on factors, e.g., choice of refractive indices and thickness of phosphor layer. In Figure 4a, we show the fraction of emitted light into guided modes as a function of position for a simple air–phosphor–glass system, similar to the setup in Figure 2. As the phosphor layer thickness increases, we notice new guided modes emerge. The sudden jumps in guided mode LDOS at certain thicknesses correspond to the cutoff conditions of the waveguide modes. For thicknesses below approximately *d*_phosphor_ ≈ 0.4 μm, only the fundamental guided mode (TE_0_, TM_0_) are supported, while higher order modes (TE_1_, TM_1_, and so on) appear just above 0.4 μm and just above 0.7 μm. The LDOS of the guided modes is highest when modes just appear. As the phosphor thickness increases, the mode confinement deteriorates, leading to a decrease in the LDOS of the guided modes until the next mode appears. In Figure 4b, we observe that the figure of merit (FOM) parameter increases as the spacer layer thickness increases. This could potentially result in a performance boost of over three times compared to the reference case. There is a wide range of refractive indices available for the phosphor films, from approximately 1.55–2.3. This range is typical of mixtures of polymer hosts with organic molecules (lower end of the range) or quantum dots (higher end of the range, at high volume fractions).^58,59^ The ideal spacer thickness, which shields the phosphor-guided mode from the GaN, depends on the choice of phosphor because a higher phosphor index and thickness present a stronger confinement. Figure 4c maps the suitable spacer thicknesses depending on the phosphor index and thickness. Here, “suitable” thickness is defined somewhat arbitrarily as the point where the efficiency versus spacer thickness has traversed 80% of its rapid upward slope (refer to Figure 3g). Finally, in Figure 4d, the corresponding potential figure of merit (FOM) for different phosphor layer thicknesses (*d*_phosphor_) and refractive indices (*n*_phosphor_) that can be achieved with such an optimized spacer thickness is shown. The distinct repeated curved features in these maps trace the appearance of new guided modes. Generally, in a practical scenario, the constituent materials determine the phosphor layer’s refractive index; using these maps, one can choose the appropriate spacer thickness to achieve the optimum enhancement. Overall, we conclude that the strategy of recuperating quasi-guided LDOS contributions in phosphors has a two- to 3-fold improvement potential for micro-LEDs over a large bandwidth of phosphor refractive indices, assuming that suitable extraction efficiencies can be devised to extract the guided mode emission.

**Figure 5:**
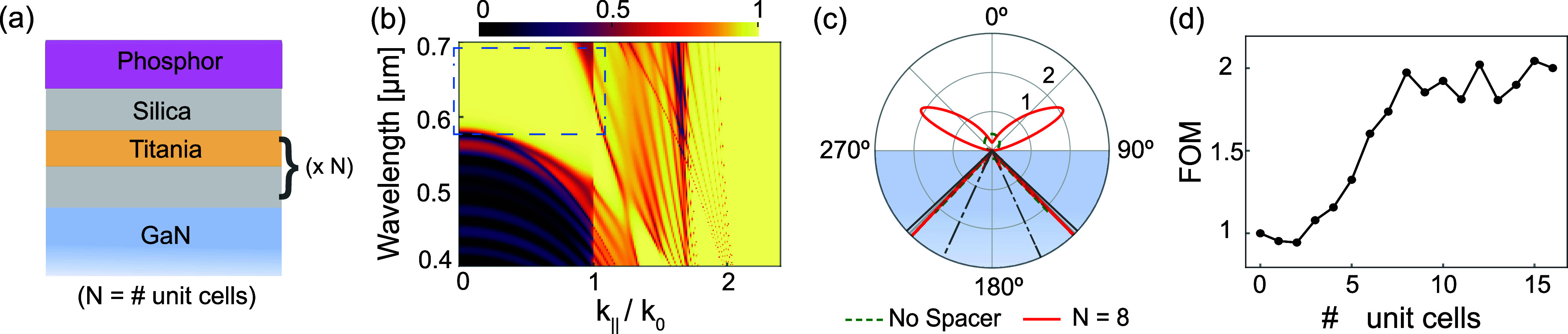
Theoretical analysis of a Bragg stack spacer design between GaN and phosphor. (a) Sketch of the system with *N* repeating alternating layers of silica (0.118 μm thick, *n* = 1.46), and titania (*n* = 2.45), forming a periodic structure of 0.189 μm period, terminated with silica between GaN and phosphor (0.4 μm thick, *n* = 1.75), capped with air. (b) Wavevector resolved reflectivity for *N* = 8 unit cells for light impinging from the GaN side. Multilayer structure is optimized for almost full reflection around the emission wavelength at high angles (blue dotted box). (c) Radiation pattern as a function of polar angle for randomly oriented dipoles, comparing no spacer (green dashed curve) to *N* = 8 unit cells (red solid curve). Upper hemisphere is magnified 20× for clarity. (d) Figure of merit (FOM) as a function of the number of unit cells (*N*), with *N* = 0 indicating no spacer.

After establishing the general principle of guided mode LDOS engineering, one might wonder if using more advanced spacer design strategies would provide real benefits. For instance, replacing the silica spacer with a dielectric multilayer with a carefully adjusted photonic stop band could prevent converted red emission from entering the GaN while still allowing blue pump photons to pass through from the GaN to the phosphor. In Figure 5, a Bragg stack concept is presented using alternating silica and titania layers in 1D photonic crystals. We assumed a fixed refractive index of 2.45 for the titania layer.^60^ The plot in Figure 5a shows the angle-resolved reflectance of the complete layer system, including the top air half space, for 8 unit cells. It consists of the Bragg stack with a silica termination layer between the phosphor and GaN layers and is illuminated from the GaN side. The quarter wave stack is designed with an optimized periodicity of 189 nm (silica thickness of 118 nm) to allow blue pump light to pass through over a wide range of propagation angles while also being strongly reflective at the emission wavelength. Additionally, we calculate the generalized reflectance for large wave vectors, including evanescent waves everywhere except in the GaN. The stack only causes a 10% reflection loss for blue pump photons, while there is a broad angular and wavelength range with almost 90% reflection in the emission wavelength band (dashed box). The emission toward the air side is significantly improved, as shown in the calculated radiation pattern (isotropic dipole average, with dipoles located in the middle of the phosphor layer) in Figure 5b with 8 unit cells (red solid curve) compared to the no spacer case (green dashed curve). We performed the comprehensive energy balance analysis, which included separating the local density of states (LDOS) into guided and propagating fractions and evaluating pump absorption, as shown in Supporting Information Figure S1. With approximately eight or more unit cells, we observe a gain from 8 to 21% in direct radiation emission toward the air side, an increase in the guided part to almost 20%, and a decrease of emission into GaN from 90 to 58%. The figure of merit (FOM) of this design, factoring in the product of absorption in the phosphor layer at the pump wavelength and emission contributions (sum of direct radiation to air and guided part), is around 2× compared to the reference case (i.e., without spacer) as shown in Figure 5d. Despite a noticeable increase in direct radiation, the complex Bragg stack does not significantly improve performance compared to the simple silica spacer design. The underlying cause is that the presence of the high index titania layer (*n* = 2.45) causes the emission from the phosphor to get trapped in those spacer layers, degrading the quality of the quasi-waveguide mode nature of the phosphor layer (see Supporting Information Figure S1d).

### Experimental Realization

**Figure 6:**
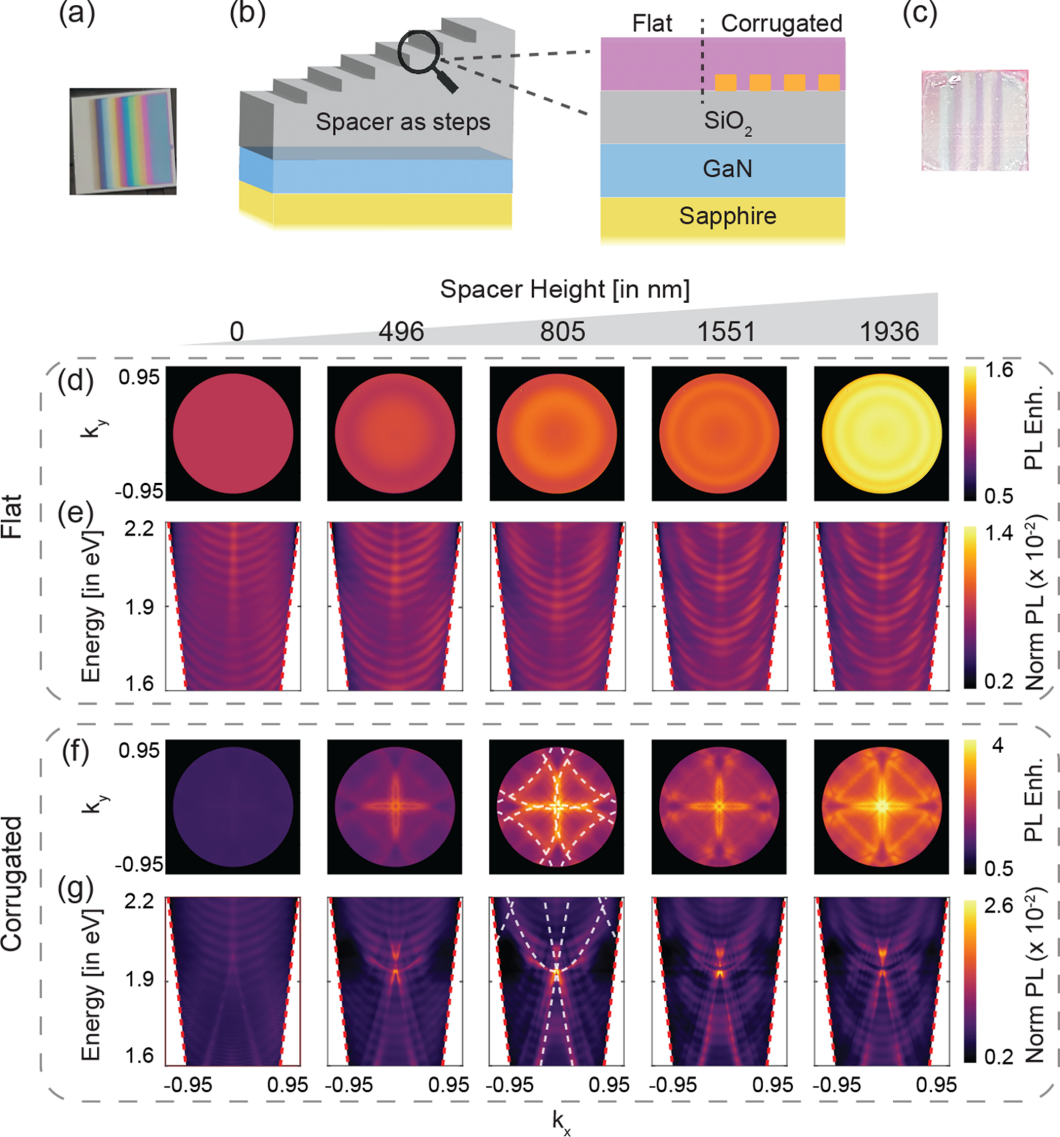
Experimental results for directional photoluminescence extraction from phosphors on GaN with varying heights of a low-index silica spacer. (a) White light image of an example staircase evaporated on silicon, showing the height differences through colors (etaloning). (b) Schematic: stepped silica spacer atop 5 μm thick GaN on sapphire, allowing multiple height measurements from a single sample. Each step includes a flat section and a corrugated section with a fabricated plasmonic lattice and is topped with a ∼400 nm thick spin-coated phosphor layer. (c) White light image of one of the samples used for measurements, showing a pink tinge from the phosphor dye. For the flat configuration, panels (c) and (d) present the back focal plane (Fourier) photoluminescence enhancement and normalized photoluminescence dispersion plots as the spacer thickness increases. Panels (e) and (f): corrugated configuration. For the 805 nm spacer thickness, the theoretical isofrequency projection of repeating circles is overlaid as dotted white lines on the dispersion relation.

In this section, we describe our experimental realization to test the hypothesis that creating and extracting a guided mode can significantly enhance the brightness of phosphor emission. According to theory, there is no clear advantage to using complex multilayer structures between GaN and phosphor. Therefore, we focused on using a simple silica spacer. Our substrate consists of a double-sided polished sapphire substrate with a 5 μm thick GaN layer, which mimics the refractive index environment of a blue LED chip. To take measurements at various spacer heights from a single sample, we deposited silica (SiO_*x*_) spacers using an evaporator with a moving shutter, which allows us to sample a range of ∼2 μm in spacer height in steps of approximately 20 nm. Figure 6a shows such an example silica staircase on a Si substrate under white light. The different thin-film interference colors directly demonstrate the spacer thickness variation. Using electron beam lithography, we embed diffractive metasurfaces in the phosphor to extract the guided emission by fabricating square-lattice plasmonic particle arrays. We provide patterning at all spacer heights while leaving an adjacent strip unaltered over the entire length of the staircase. This allows us to compare light extraction performance between flat and corrugated regions at the same spacer heights under similar processing conditions. Finally, we apply a roughly 400 nm thick polystyrene layer on top using spin coating. The polymer layer is doped with 2 wt % of a perylene-based commercial dye called Oracet FL Red 305, BASF (previously known as Lumogen Red), and acts as the phosphor layer. A schematic of the resulting sample geometry is displayed in Figure 6b. The perylene dye has a broad bright photoluminescence (PL) spectrum from approximately 560–750 nm (see Supporting Information Figure S2a) and is specifically designed and marketed for long-term photostability at high excitation rates and thermal loads in lighting conditions.^61^ The relatively low dye concentration prevents interference between the dye molecules’ resonance and plasmonic-waveguide coupling while ensuring sufficient intensity for probing near-field coupling of various optical modes.^34,53^ Previous studies confirm that the absolute Purcell effect is typically insignificant for this dye and class of periodic metasurfaces. Since we work with a high-efficiency emitter, there is, in any case, no brightness gain that can be reached with absolute Purcell enhancements. The dye’s near-unity QE remains largely unaffected despite the plasmonic antennas, as shown in prior near-field super-resolution microscopy studies of similar metasurface systems (without GaN underneath).^62,63^ This is attributed to the fact that most dye emitters are sufficiently distant from the metal to minimize quenching effects.

For this layer thickness, the phosphor layer acts as a waveguide with fundamental TE and TM modes (calculated average mode indices *n*_WG_ ∼ 1.53 at λ = 0.6 μm^64^) in a sandwiched geometry between air and glass (*n* = 1.46) super(sub)strates. To extract the energy of the waveguide mode in the forward direction, we use a simple array of square particles with a pitch of 0.42 μm. This pitch corresponds to the second-order Bragg condition for the waveguide mode around the maximum emission wavelength of the dye. The chosen particles are Ag nanocylinders with a diameter and height of 100 and 40 nm, respectively. The plasmon resonance is around 650 nm, which overlaps with the dye PL band and is consistent with previous related research works. We used Fourier microscopy mode in an inverted fluorescence microscope set up to directly measure the angle-resolved photoluminescence from the sample at all angles within the numerical aperture of the objective (NA = 0.95, up to θ ∼ 72^0^ in air). As a pump source, we use a continuous-wave laser at 405 nm at normal incidence with an approximate diameter of 75 μm at the sample plane and an incident power of around 8 mW. We examine the emission from the phosphor/dye side and block the pump light by combining a long-pass filter and dichroic mirror. The integrated counts on a charge-coupled device (CCD) camera are typically on the order of 10^9^ with 150 ms exposure time. The 2D back focal plane images that we present are panchromatic images. To map out the dispersion diagram, we image a slice of the Fourier image centered at *k*_*x*_ = 0 onto the slit of an imaging spectrometer.^65^ For panchromatic Fourier images, we normalize data to obtain photoluminescence enhancement (PLE) by dividing it by the corresponding images measured from the same layer but directly on top of the GaN layer. The dispersion images are expressed as a normalized photoluminescence quantity by dividing the raw dispersion image by the measured spectrum shape of the dye. We do this to adjust for the variation in the dye’s photoluminescence intensity spectrum to understand the behavior of the spacer and plasmonic array in isolation. Figure 6 presents data for select salient spacer heights. We refer to the Supporting Information for all 41 spacer heights that we probed (from 0 to 180 nm height in 21 nm steps and subsequently up to 2.1 μm in 62 nm steps). First, let us consider unpatterned samples where the spacer height varies, but no particles are present. The Fourier images of PLE (Figure 6c) display concentric rings, with the number of rings increasing as spacer thickness increases. There is a modest enhancement in emission intensity at large spacer heights. In spectrally resolved Fourier images (Figure 6d), we observe a constant number of closely spaced (0.02 eV) parabolic bands that are common to all spacer heights, along with broader parabolic bands on top of them. The narrow fringes are due to Fabry–Perot interference in the 5 μm GaN layer. The broader parabolic bands arise from Fabry–Perot resonances in the spacer layer. These bands are particularly noticeable for the thicker spacer layers (e.g., 1.5 μm spacer/∼0.22 eV spacing between bands), and the spectral spacing decreases with increasing thickness. The concentric bands in the panchromatic Fourier images are due to these spacer resonances.

Having established the basic behavior of the stratified system, we now discuss the performance of the corrugated samples. In Figure 6e, the Fourier back focal images generally show the appearance of multiple intersecting circles, a familiar occurrence in the field of controlling fluorescence with plasmonic lattices.^66,67^ The choice of periodicity ensures intersection near **k**_∥_ = 0, designed for strong outcoupling of the guided mode LDOS contribution in the forward direction. Notably, the features are essentially nonexistent when the spacer height is zero, but they become significantly more intense as the spacer thickness increases. This observation aligns with the prediction that the quasi-guided mode in the phosphor layer requires a minimum spacer thickness of several hundred nanometers to develop, which is a necessary condition for promoting increased emission into the waveguide mode with high in-plane momentum magnitude (|*k*_∥__,WG_|/*k*_0_ = *n*_WG_). This quasi-guided mode is directionally coupled out through the periodic corrugation, which provides the required reciprocal lattice vectors to diffract the guided mode emission into the light cone according to the equation **k**_∥__,out_ = **k**_∥__,in plane_ + **G**. Here  with the integers *m*, *n* representing the order of diffraction. At a spacer thickness of 805 nm, the diagram confirms this concept. Dashed circles overplot the waveguide dispersion shifted by *n*, *m* = −1, 0, 1 and assuming a mode index *n*_WG_ ∼ 1.53, for the central emission wavelength at λ ∼ 0.6 μm. The correspondence with the observed features is excellent. However, the measured features appear blurred due to spectral averaging over the fluorescence bandwidth of approximately 150 nm. Once the Fourier-space data is spectrally resolved (Figure 6f), the folded waveguide dispersion appears as sharp, bright diagonal lines, and the parabola intersects at *k*_∥_ = 0.

The linear dispersion bands represent the waveguide mode dispersion folded back from the [±1, 0] reciprocal points, while the parabolic bands derive from [0, ±1] diffraction. These general observations align with reported measurements on plasmon lattices for fluorescence. The diffracted orders are absent at zero spacer height owing to the absence of the phosphor-guided mode. At spacer heights above 500 nm, the photoluminescence enhancement is significantly higher than that achieved in the noncorrugated sample, confirming the main hypothesis that creating a guided mode and then extracting it is a fruitful design strategy. A more subtle effect is that the sharp, bright bands resulting from band folding of the quasi-guided emission overlap with the Fabry–Perot interference bands that occur in the multilayer stack even in the absence of the plasmon particle lattice. This overlap should not be seen as a simple incoherent summation of intensities. For instance, there are clearly noticeable sharp, dark diagonal lines in the measured band structures (see, e.g., Figure 6f for the 805 nm case), which correspond to dark replicas of the bright folded dispersion relation (dashed white line). We attribute these lines to interference between emission channels that directly couple out from the quasi-guided mode and paths that couple out after multiple reflections in the layer stack. A close examination also shows dark diagonal lines in the 2D Fourier images (e.g., Figure 6e, dark diagonals converging on *k* = 0 for the 805 nm case). These dark features are highly surprising and have not been reported in the field before. They are somewhat reminiscent of Kossel and Kikuchi lines in the sense that they are destructive interference features that appear in an incoherent/partially coherent signal.^68−70^ The detailed explanation of these features is beyond the scope of this paper.

**Figure 7:**
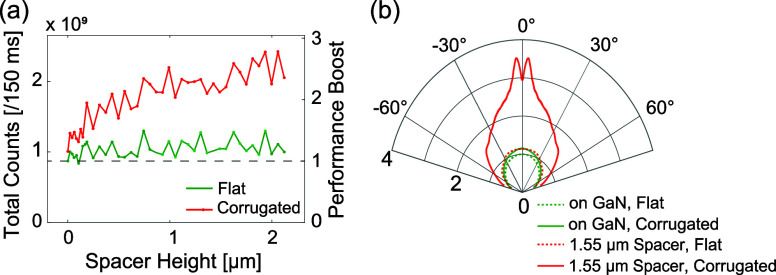
Performance comparison as a function of spacer height. (a) Total photoluminescence counts plotted against spacer thickness for flat (green) and corrugated (red) cases. The right vertical axis indicates the performance boost, with the black dashed line at unity representing the reference case (no spacer, no corrugation), i.e., phosphor directly on top of blue GaN die. (b) Azimuthally averaged directional photoluminescence enhancement plotted against the polar angle for various configurations. For the flat configuration, green dashed and solid curves denote cases with no spacer and a 1.55 μm spacer, respectively. Red dashed and solid curves represent the same spacer conditions for the corrugated configuration.

From a performance viewpoint, the crucial question is whether the combination of dielectric spacer and metasurface between GaN die and phosphor actually enhances the overall conversion of blue-to-red light. We evaluate this by measuring the brightness from real space photoluminescence (PL) intensity maps that we integrate over the entire area and plotting as a function of spacer height (see Figure 7a). The measurements on the flat configuration suggest that compared to a phosphor directly on GaN (green curve), there is either no benefit or, at most, a very modest benefit from introducing a spacer. The presence of the diffractive lattice corrugation (red curve) results in a partial extraction of the emission into the phosphor-guided mode. To quantify the performance improvement (right hand *Y*-axis in Figure 7a) from the spacer, both curves are normalized to the reference configuration (no spacer, no lattice corrugation). When the phosphor with the diffractive lattice is directly placed on top of GaN, there is no performance improvement. For spacer heights greater than 1.2 μm, the metasurface configuration shows a performance boost of at least 2.5 times, whereas the flat case with a spacer alone results in an increase of ca. 1.2. We note that the apparent ‘noise’ in the collected counts versus spacer height is not, in fact, noise but is largely due to the intrinsic thin-film interference in the device stack that varies with the thickness variation of the spacer (also observed in the theoretical calculation, refer Figure 3h and note that flat and corrugated systems show correlated fluctuations). Our theoretical calculations with normally incident pump light reveal a sinusoidal thin-film interference pattern, resulting in a ∼20% variation in absorbed power around the mean value. In Supporting Information Figure S3, we plot the horizontally binned spectrum obtained from the dispersion images demonstrating similar brightness enhancement. We average the back focal plane images over the azimuthal coordinate to quantify the directionality of emission. Polar plots of the azimuthally averaged emission radiation patterns are presented in Figure 7b. For the reference configuration, i.e., in the absence of spacer and corrugation, the photoluminescence directionality enhancement (PLDE) factor is 1 (as shown by the green dashed curve). Both for the case of an inserted spacer (1.55 μm) but no corrugation (red dashed curve) and for the case of a corrugation without spacer (green solid curve), the PLDE is only marginally improved over the reference case. This suggests that the diffractive metasurface alone is ineffective by itself and only provides benefit in conjunction with the spacer that recovers the phosphor’s quasi-guided mode. Indeed, at 1.55 μm spacer and corrugation, we find a significant increase of the PLDE in the normal direction by approximately four times (red solid curve). The experiments were conducted using collimated laser excitation at 405 nm wavelength and normal incidence, differing in directionality and wavelength from the Lambertian 450 nm pump light assumed in the theoretical calculations, which is typical of LEDs. As regards the wavelength difference, based on theoretical calculations (as our design study) and accounting for the low dispersion of GaN and SiO_2_, in a Lambertian pump LED scenario, a wavelength shift from 450 to 405 nm is not expected to change performance metrics, aside from a concomitant reduction in optimal spacer thickness by ∼10%. Regarding the angular distribution, we choose to experiment with collimated incidence as there is only a minor variation with spacer thickness in absorption (ca. 20%). Thereby, the experiment particularly singles out the spacer strategy, i.e., creating guided modes as a prerequisite for enhanced outcoupling.

## Conclusions

We have established that more efficient driving of phosphors in microLED applications can counterintuitively benefit from strategies that promote light emission into guided modes. Our study, which is based on LDOS engineering in stratified systems, has revealed that the inclusion of a low-index spacer between a phosphor and the high-index GaN layer can ensure that a significant portion of emission is captured into quasi-guided modes propagating within the phosphor. There is a potential for a two to 3-fold increase in brightness if the guided emission is extracted, even when accounting for the fact that promoting the existence of the guided mode reduces the coupling of blue pump photons from the GaN into the phosphor. We have successfully demonstrated this design philosophy experimentally. Our results show that creating a quasi-guided mode and outcoupling it with a simple plasmonic antenna lattice leads to a 2.5-fold increase in photoluminescence. The resulting emission is highly directional, with a 4-fold enhancement toward the normal. This paper thus shows that (i) indeed, creating quasi-guided modes in the phosphor can lead to brighter emission, while (ii) the proven strategy of plasmonic surface lattice resonance metasurfaces is very effective also in such a complicated stratified system, although the stratification does result in interference phenomena between Fabry–Perot fringes and lattice resonance features.

The design philosophy we propose is compatible with a wide range of phosphors, and proposed materials for various spacer geometries are highly compatible with current industrial deposition processes,^71^ patterning methods (e.g., nanoimprint lithographies or template stripping^72−77^), and e.g., inkjet deposition of fluorophores.^78,79^ We focused our study exclusively on submicron-thick phosphor layers, which will make it impossible to reach full blue pump light absorption and efficient conversion even at very dense emitter packings. Our approach can be adapted to vertically stack multiple submicron quasi-guiding phosphor layers to achieve the desired cumulatively thicker phosphor layer.^80^ A major challenge for displays is that if a significant fraction of blue photons are not absorbed, they need to be filtered out so as not to affect the color purity. An approach that fits our framework is to integrate the phosphors with dielectric claddings such as distributed Bragg reflectors that prevent leakage of blue light^31^ and at the same time may even add control over the etendue of emission^81^ (see Supporting Information Figure S3 for theoretical calculations on a similar Bragg stack cladding layer in addition to a low-index silica spacer layer).

From the metasurface design perspective, there are many interesting questions. There is scope for even larger enhancements by simultaneously engineering absorption improvements. If instead of a Lambertian photon distribution, the blue pump LED were engineered itself for higher directivity, the total internal reflection effect that blocks blue light from reaching the phosphor could be removed, while a narrow wavevector distribution could also enable more effective diffractive absorption engineering in the phosphor. Such directive blue LEDs have been reported recently based on nanophotonic engineering^82^ while also geometrical features such as back reflectors or pyramidal sidewalls could be used.^83^ While we deployed an unoptimized, commonly used plasmon lattice as an extraction strategy, the proposed spacer geometry is compatible with many different nanophotonic strategies^36^ that may thus become applicable to the highly relevant but photonically disadvantageous microLED environment. A very interesting design challenge is posed by the finite lateral footprint of micro LEDs: while we used periodic scatterer arrays for small lateral devices, it is likely that different, aperiodic patterns can outperform them. Numerical techniques^84−86^ will need to be extended to jointly optimize the design parameters of the corrugation and spacer layered geometry to deal with the combined challenges of absorption enhancement, quasi-guide mode engineering, and optimal extraction patterns tailored to the phosphor and lateral footprint.

## Methods

### Calculation of Quasi-Guided LDOS Contribution

The emission of a dipole into a quasi-guided mode in the phosphor is characterized by the presence of a distinct sharp peak in the wavevector resolved LDOS integrand curve within the range *n*_silica_*k*_0_ (1.46) < *k*_∥_ < *n*_phosphor_*k*_0_ (1.75). This peak is a true pole for a truly guided mode, whereas, for a quasi-guided mode, the leaky nature causes a broadened contribution of finite width and height. We define the ‘quasiguided’ contribution to the emission through the following procedure. First, we identify the mode indices of the quasi-guided modes (*n*_q-g_) by examining the *u*-values at which the LDOS integrands strongly peak. Next, we identify these contributions in the far-field radiation patterns. Since the GaN substrate has a higher index, the quasi-guided modes radiate into the GaN into sharply defined peaks at angles that derive directly from the mode indices via sin^–1^(*n*_q-g_/*n*_GaN_). We define the quasi-guided LDOS contribution as the difference between the total LDOS and the total radiated LDOS, where we define total radiated LDOS as the integrated radiation pattern excluding the sharp quasi-guided mode contributions. The exclusion uses a somewhat arbitrary criterion, as we exclude a mode index range of 10^–4^ around the quasi-guided mode peaks.

### Fabrication Steps

We procure sapphire substrates with 5 μm of GaN on top from University Wafer. This GaN-coated substrate mimics the refractive index environment provided by a blue LED. We used two fabrication steps to create spacers with a thickness of up to 2.1 μm. First, we coated the substrates with a silica spacer of a fixed thickness (a multiple of 0.5 μm) using high-density plasma of N_2_O and SiH_4_ gases in an evaporation chamber (Oxford PlasmaPro 100 ICPECVD). Then, we used a linear moving shutter to fabricate a step-and-terrace spacer layer via electron beam physical vapor deposition (Polyteknik Flextura M508 E), followed by metasurface fabrication on a portion of each terrace. The maximum height difference across the sample is approximately 600 nm, distributed laterally over a distance of about 12 mm on a 15 × 15 mm^2^ substrate in 10 steps (∼60 nm step height and ∼1.2 mm step width). Due to the shadowing effect of the shutter, the steps are slightly graded rather than abrupt, resulting in a wedge-like structure with a minimal slope. These gentle undulations have negligible impact on the spinning of the resist layer, and the e-beam lithography writer follows the height profile for focusing, using a laser for height control. The following steps were pursued to fabricate the corrugation: we spun 150 nm PMMA 495-A8 resist on the substrates and deposited a 20 nm layer of Germanium (Ge) through thermal evaporation to serve as a hard mask. After that, ca. 60 nm CSAR AR-P 6200:09 resist was spun and then exposed (Raith Voyager 50 keV), with a nominal dose of 130 μC/cm^2^. After developing the resist layer, we etched the Ge and PMMA layers using SF_6_ and O_2_ plasma. Next, we evaporated 40 nm Ag and performed a liftoff process in warm acetone. The lateral dimension of the arrays was 100 μm × 100 μm each. Supporting Information Figure S2b shows a scanning electron microscope (SEM) image of one such metasurface array. Finally, we spun a 0.4 μm thick polystyrene-based polymer film (doped with 2 wt % BASF-Oracet FL Red 305 dye in toluene solvent), which functions as the phosphor layer. We have provided a schematic flowchart of the fabrication process described above in Supporting Information Figure S5.

### Optical Characterization

We used a custom-built inverted microscope where the pump and detection both go through the same objective (Zeiss N-Achroplan 63×, 0.95 NA, coverglass = 0) and come from the phosphor (dye) side, as illustrated in Supporting Information Figure S4. We use a 405 nm continuous wave (CW) laser (Cobolt 06-MLD) to excite the sample. The setup used is similar to as described in this literature.^65^ We use an epi lens in the pump path to achieve a beam diameter of approximately 80 μm on the sample plane. The incident pump power is measured as 8 μW at the sample plane using a photodiode (after the objective, Thorlabs S121C power sensor). After that, we filter the fluorescence using a long pass filter (Chroma, ET425lp) following the dichroic mirror (Chroma, ZT405rdc-UF1-25 × 36) to eliminate any unwanted reflected pump light. The fluorescent light is focused onto a thermoelectrically cooled Si CCD camera (Andor Clara) or a spectrometer (Shamrock 303i spectrometer with a slit opening of 50 μm) with a (Andor Ivac) Si CCD detector using a tube lens of 200 mm after being reflected by a mirror. We image the objective’s back focal plane onto the CCD camera by flipping a Fourier lens. We set the exposure time to 150 ms for the Andor Clara and 2 s for the Andor Ivac camera.

## Supporting Information Available

Theoretical design calculations of Bragg stack spacer; spectrum of dye-doped polymer layer acting as the phosphor layer in the experiments; scanning electron microscope (SEM) image of a metasurface array; horizontally binned experimental spectra comparison for different configurations; theoretical design of Bragg stack cladding layer on top of the phosphor layer for reduction of blue photons leak with the spacer layer; sample fabrication process schematic flowchart; schematic of the experimental setup; detailed file references for measurement observations across all spacer heights (pdf); and measurement observations (Fourier and normalized dispersion photoluminescence enhancement) for all spacer heights with and without corrugation (mp4), organized in a separate dedicated folder named Observations (PDF)
Observations of Fourier. dispersion, Fourier spacer, and dispersion spacer (ZIP)
