# Supplementary material for: Metasurface-Based Phosphor-Converted Micro-LED Architecture for Displays—Creating Guided Modes for Enhanced Directionality
Source: ACS Nano. 2024 Dec 23;19(1):1238–50. doi: 10.1021/acsnano.4c13472 (PMC11752504; doi:10.1021/acsnano.4c13472)
Supplement: Supplementary file 1 — nn4c13472_si_001.pdf [file nn4c13472_si_001.pdf]

Supplementary: Metasurface-Based Phosphor-Converted Micro-LED Architecture for Displays – Creating Guided Modes for Enhanced Directionality

Debapriya Pal[a],[1] Toni López,[2] and A. Femius Koenderink[b1]

[1]*Department of Physics of Information in Matter and Center for Nanophotonics, NWO-I Institute AMOLF, Science Park 104, NL 1098XG Amsterdam, The Netherlands*

[2]*Lumileds Germany GmbH, Philipsstr. 8, D-52068 Aachen, Germany*

(Dated: December 7, 2024)

CONTENTS

I. Bragg stack spacer S2

II. Phosphor spectrum & Scanning electron microscopy S3

III. Experimental Spectra comparison S3

IV. Bragg stack cladding layer for the reduction in the leak of blue photons S4

V. Sample Fabrication S5

VI. Experimental setup S6

VII. Observations for all spacer heights S7

[a] Corresponding author: d.pal@amolf.nl

[b] Corresponding author: f.koenderink@amolf.nl

I. BRAGG STACK SPACER

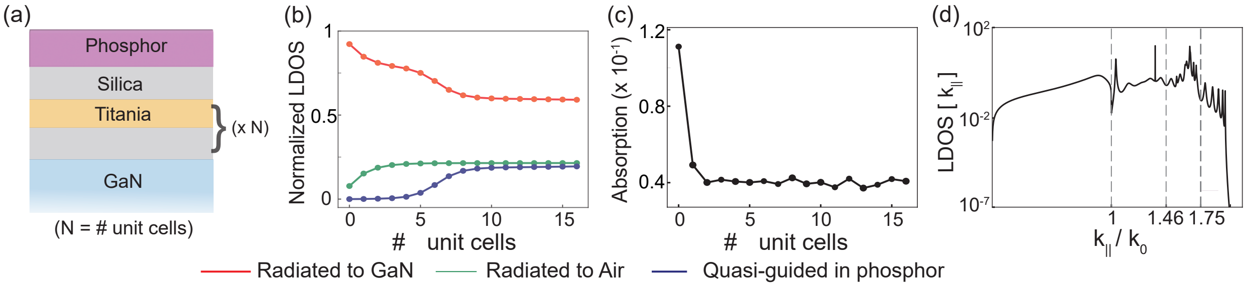

FIG. S1. Concept of a Bragg stack design of spacer layer and theoretical analysis. a) Sketch of the system consisting of GaN (substrate, n = 2.4), N repeating alternating layers consisting of silica (0.118 μm thick, n = 1.46), and titania (n = 2.45) with a periodicity of 0.189 μm with silica as termination layer, phosphor (0.4 μm thick, n = 1.75) with air (n = 1) as super substrate. As a function of the number of unit cells, plots of b) the emission contribution (normalized LDOS) of the phosphor layer into different channels and c) the absorption of blue photons. In plot b), the red, green, and blue curves represent radiation to the GaN, airside, and quasi-guided into the phosphor layer, respectively. d) Wave-vector resolved power emission plot by a randomly oriented dipole at the middle of the phosphor layer for N = 8 unit cells. Here, N = 0 means there is no spacer or reference configuration.

In the absence of a spacer (N=0, reference case), only 8% of the light emission is directed towards the air side, while the majority of the emission (92%) is directed towards the GaN side as plotted in Figure S1b. With N = 8 unit cells, the proportion of emission directed towards the air increases to about 22%. This differs from a typical silica spacer, where the increase in spacer thickness does not significantly improve the direct emission toward the air. As the number of unit cells increases, there is a higher fraction of emission in quasi-guide modes in the phosphor layer, with a decrease in emission towards the GaN (60%). The absorption of blue photon flux reduces by a factor of 2.6x (Figure S1c). As discussed in the main manuscript Figure 5d, the figure of merit (FOM) for this stack design is only around 2x compared to the reference case. The performance of this complex design stack is not high compared to the normal silica spacer, even though there is a gain of around 2.75 times in direct radiation towards the air side. Figure S1d shows the power emitted by a randomly oriented dipole in the middle of the phosphor layer for N = 8 unit cells. The

range $n_{\mathrm{silica}}k_0\,(1.46) < k_{||} < n_{\mathrm{Phosphor}}k_0\,(1.75)$ corresponds to light that may propagate in the phosphor layer. The absence of sharp peaks here indicates the degradation of the quasi-waveguide nature of the phosphor layer in contrast to the silica spacer case Figure 3d. The presence of peaks beyond (>) $n_{\mathrm{Phosphor}}k_0\,(1.75)$ means emission is into modes that propagate into GaN and high index titania spacer layers and evanescent in the air, phosphor, and silica layers.

II. PHOSPHOR SPECTRUM & SCANNING ELECTRON MICROSCOPY

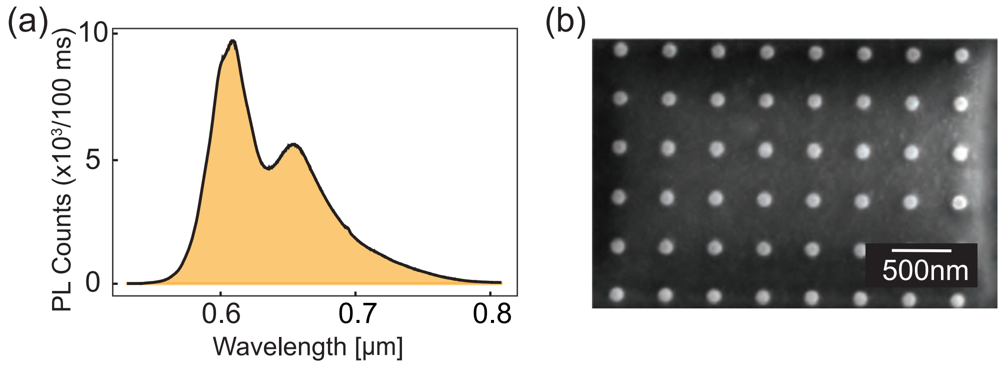

FIG. S2. Dye spectrum and SEM. a) The photoluminescence spectrum is from the phosphor layer that consists of a 400 nm dye-doped polymer layer. b) Scanning electron microscope image of fabricated Ag nanocylinders of height 40 nm and diameter 100 nm arranged in a square array with a pitch of 420 nm fabricated using an E-beam lithography process.

The photoluminescence spectrum of the 2 wt% Oracet Red F305 dye-doped polymer (polystyrene) layer, which serves as the phosphor layer for the experiments, is plotted in Figure S2a. Figure S2b shows a scanning electron microscope image of one of the plasmonic particle arrays. After the liftoff process, around 20nm Electra Espacer layer is applied for SEM imaging (5 kV). We removed the Espacer layer by rinsing the sample twice in water before spinning and coating it with the dye-doped polymer layer.

III. EXPERIMENTAL SPECTRA COMPARISON

In Figure S3, we present the horizontally binned dispersion image obtained by summing over the wave vector for easier interpretation. The data show no significant improvement

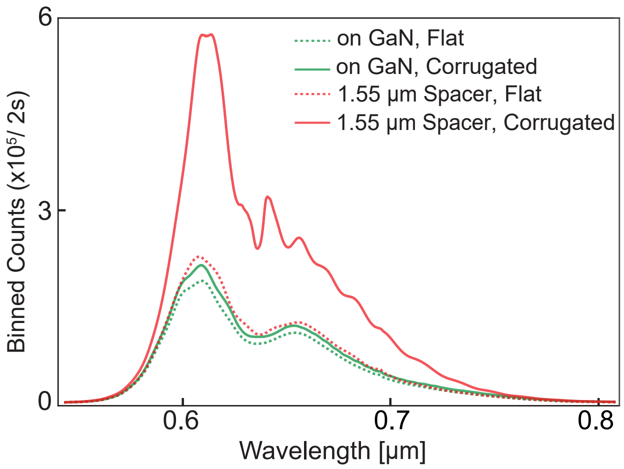

FIG. S3. Performance comparison via spectrum for different configurations.

when the metasurface is directly on GaN. However, a 1.55 μm thick spacer layer results in a similar 2.5x emission enhancement.

IV. BRAGG STACK CLADDING LAYER FOR THE REDUCTION IN THE LEAK OF BLUE PHOTONS

For a 400nm thick phosphor layer with an absorption coefficient of 0.01, only about 10% of blue pump photons are absorbed in a single pass, and around 80% passes through to air. We present in Figure S4 a distributed Bragg reflector on top of the phosphor, also called a cladding layer, to act as a color filter. The simple quarter-wave stack is designed with an optimized periodicity of 115 nm (silica thickness of 72 nm) to allow converted red photons (= 0.6 μm) to pass through to air while also being reflective at the blue pump wavelength. This stack with N = 8 unit cells and 1 μm thick silica spacer reflects 65% of the blue pump photons (= 0.45μm) and results in only around 10% reflection loss around emission band wavelength, as shown in angle-resolved reflectance plot of Figure S4b. The figure of merit (FOM) is plotted for two cases - no cladding layer (red dashed line) and with N= 8 unit cells Bragg stack cladding layer (blue solid line) as a function of silica spacer thickness, d_{spacer}, in Figure S4. When a cladding layer is added to the system, there is a 1.4x improvement in the Figure of Merit (FOM) compared to the scenario without a cladding layer but with a finite spacer layer. The minimum spacer thickness required to achieve similar performance is also reduced. In addition to reducing blue photon leakage, the FOM improves because

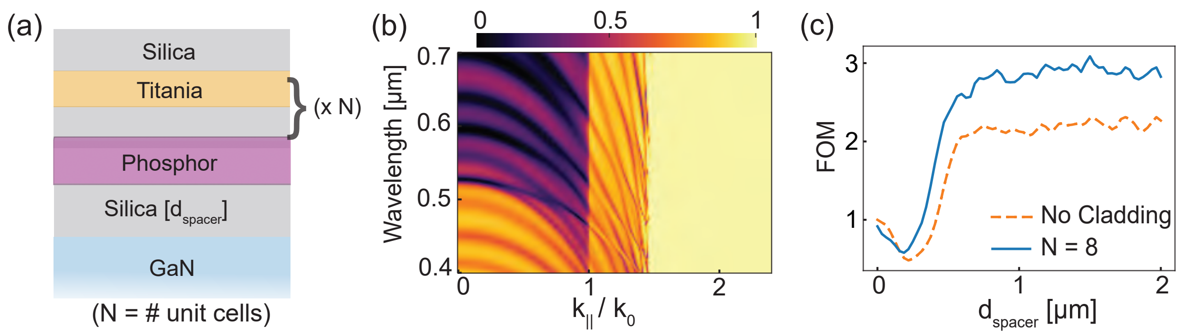

FIG. S4. Concept of Bragg stack as cladding layer in addition to silica spacer layer and theoretical analysis. a) Sketch of the system consisting of GaN (substrate, n = 2.4), silica spacer (d_{spacer} thick), phosphor (0.4 μm thick, n = 1.75), N repeating alternating layers consisting of silica (0.072 μm thick, n = 1.46), and titania (n = 2.45) with a periodicity of 0.115 μm with silica as the termination layer on top of phosphor, with air (n = 1) as super substrate. b) Angle-resolved band structure with N = 8 unit cells in terms of reflectance as a function of wavelength for light impinging from the GaN side. The multilayer structure is optimized for almost full transmittance around emission wavelength (= 0.6 μm) but considerable reflection for blue pump photons (= 0.45μm). c) Figure of merit (FOM) as a function of silica spacer (d_{spacer}) thickness with no cladding (red dashed line), and N = 8 Bragg stack (blue solid line) on top of phosphor.

the quasi-waveguide mode of the phosphor layer is enhanced by symmetrizing the index distribution on top instead of having it exposed to air.

V. SAMPLE FABRICATION

In Figure S5, we outline the fabrication process of our sample: Step (a) - The double-polished 5 µm GaN on sapphire substrate is cleaned using sonication followed by O_2 plasma cleaning. Step (b) - A silica spacer layer is deposited via ICPECVD, with step-and-terrace features added using a linear shutter in an E-beam evaporator. (c) We prepare a stack of 150 nm PMMA, 20 nm Ge, and 55 nm CSAR for lithography. The design is patterned on half the sample using a RAITH Voyager 50 KeV e-beam writer. (d) The exposed regions are developed to reveal the patterned areas. (e) Dry etching transfers the pattern through the Ge and PMMA layers. (f) A 40 nm silver layer is evaporated onto the sample. (g) We perform liftoff in warm acetone to remove unwanted layers and get our metasurface on

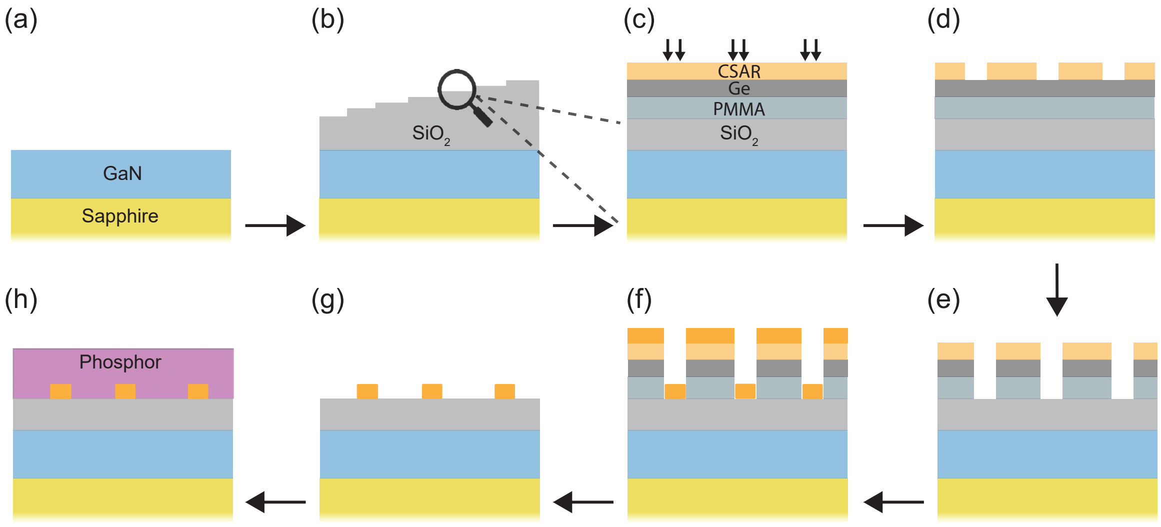

FIG. S5. Flowchart of the fabrication steps illustrated with vertical cross-sectional schematics of the sample at each stage.

the spacer. (h) Finally, a dye-doped polymer layer is spun onto the sample, serving as the phosphor layer.

VI. EXPERIMENTAL SETUP

The setup consists of an inverted fluorescence microscope. The sample is illuminated from the air side with a pump laser, and PL emission is collected in the same direction as the pump and through the same objective. The simplified schematic in Figure S6 illustrates a continuous wave (CW) laser emitting light at 405 nm. An epilens is employed to focus the excitation light onto the objective's back focal plane to provide wide excitation illumination at the sample plane. The resulting excitation spot is around 75 μm in diameter. The fluorescence is collected and, after passing through the dichroic mirror, it is focused into a detector, which can be either a camera or a spectrometer, using a tube lens. A Fourier lens can be flipped in to map the objective's back focal plane (denoted by the black dotted line) onto the detector.

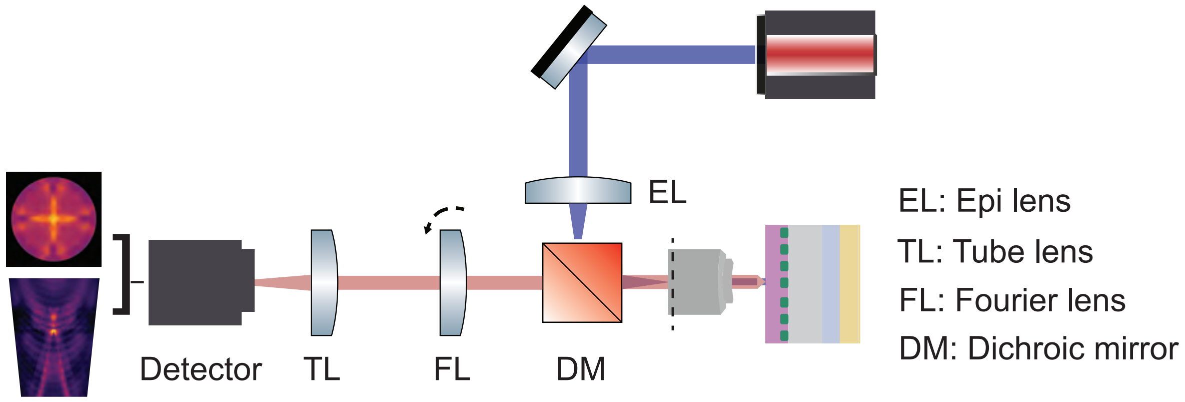

FIG. S6. Simplified schematic representation of the experimental setup used for measurements.

VII. OBSERVATIONS FOR ALL SPACER HEIGHTS

The measurement observations for all spacer heights, both with and without corrugation, are presented in .mp4 files in the Observations folder. Figure 6 in the main text showcased data for a selected few spacer heights. The file names and their corresponding data are as follows:

- 1_Fourier_OnlySpacer: Fourier data with spacer only.
- 2_Dispersion_OnlySpacer: Dispersion with spacer only.
- 3_Fourier_Spacer_Corrugation: Fourier data with spacer and corrugation.
- 4_Dispersion_Spacer_Corrugation: Dispersion data with spacer and corrugation.
